# Death from Cardiac Neuralgia

**Published:** 1867-05

**Authors:** Edward Warren

**Affiliations:** Professor of Surgery, Washington University, Baltimore, Md.


### ARTICLE V.

*Death from Cardiac Neuralgia.* Reported by EDWARD WARREN, M.D., Professor of Surgery, Washington University, Baltimore, Md.

MISS Elizabeth J. Jones, a lovely and interesting young lady of this city, aged eighteen, had been under my professional care, at various times, for the last twelve months.

During that period she had been the victim of repeated nervous attacks of an extraordinary character. These "attacks" were induced by the slightest cause which disturbed either her mental, moral or physical organization; and, alike in the violence of the hysterical symptoms by which they were characterized, and in the persistence with which they resisted treatment, exceeded anything of the kind within the compass of my observation. Accompanying these paroxyms, certain distressing neuralgic symptoms, connecting themselves with the uterus and the heart, were developed, for the relief of which chloroform was successfully employed on many occasions.

These neuralgic phenomena invariably disappeared with the subsidence of the hysterical "attacks," leaving no structural or organic lesion in either of the organs referred to, which the most thorough and accurate examination could detect.

Indeed, in the intervals between these "nervous attacks"

the uterus performed its functions with remarkable fidelity, while no unusual sound or movement disturbed the physiological monotony of the heart's action.

The last of these "attacks" preceding the one from which death resulted, occurred in February last, and was particularly violent and difficult of control.

She complained frequently of an acute pain in the heart, together with all the symptoms to which the term Angina Pectoris is applied by the authorities. After attempting a multitude of expedients, including the hypodermic use of morphia, I was constrained to employ chloroform, as had been repeatedly done before, until complete anæsthesia was produced.

Not an unfavorable symptom occurred, and the patient was not only greatly relieved, but really much benefitted by the remedy.

On the 27th of March last, she called at my office, and requested me to accompany her to the office of Professor Gorgas so as to be present and administer chloroform for the extraction of a tooth.

Having endeavored to dissuade her from taking the anæsthetic for so simple an operation, and finding that her mind was made up in this regard, and that her friends were fully aware of her intentions, I thought it my duty, at least, to see the dentist with her.

On the succeeding morning, I accompanied her to the office of Dr. Gorgas, and there learned from him that he had been unwilling to administer chloroform because of the supposed existence of some *serious organic disease of the heart.*

Having explained to *him* that the disease was nervous and not organic, that chloroform had been frequently used in her case not only with impunity but with positive benefit, and that there was, in reality, less danger to be apprehended in that instance than under ordinary circumstances, I then attempted to persuade *her* not to use the agent for so simple an operation as the one proposed.

This advice was given—not because even ordinary *danger* was apprehended in administering chloroform to one who had taken it so frequently with perfect safety—but upon the general principle that so simple and expeditious a proceedure as the extraction of a tooth does not ordinarily justify the taking of any risk whatever, for the purpose of avoiding the pain incident to it. As a means of frightening her out of her determination, when argument and persuasion had both failed me, I exaggerated the dangers of inhaling chloroform in the semi-erect posture, and insisted that she should not incur them.

As a compromise, "Richardson's Spray Apparatus" was finally used, but the taste and smell of the ether proving unpleasant, she again insisted upon the chloroform, declaring that *she would take it if it killed her*, and protesting *that if her regular medical attendant declined to administer it, she would have it done by some one else.*

Finding that she was irrevocably determined to take the chloroform, and, having presented all the difficulties and dangers of such a step fairly to her mind, I concluded that my professional obligations in this connexion, had been fully met, and that it was my duty to see that the anæsthetic was properly administered.

Taking the precaution to approximate her position as near as possible to recumbency, to open her dress, to give her whiskey and water in advance, and to keep my hand constantly on her pulse, I proceeded to administer the chloroform from an ordinary towel so arranged as to admit the *free* ingress of atmospheric air into her lungs.

She was readily impressed by the agent, the tooth was rapidly extracted, and she speedily recovered sufficient consciousness and power of locomotion to walk into an adjoining room. Full anæsthesia was not produced, for she remembered what had been said and done while apparently insensible, and chatted laughingly about it before she left the dental chair. Her pulse remained so quiet, and her respiration was so slightly disturbed during the whole period of slumber, as to induce me to call the especial attention of Dr. Gorgas to the tranquility with which these functions were performed.

After remaining about half an hour upon the sofa in an adjoining room, talking pleasantly with her companion and myself, she got up, washed her face, adjusted her hair and insisted on visiting a relative on Preston street, some mile and a half distant. To my remonstrances against the imprudence of leaving the office so speedily, and walking at all, she replied that she was "perfectly well," and went skipping gaily out of the door in advance of me.

She then took my arm and walked about half a mile, to her mother's house, on Calvert street.

Upon reaching home, she appeared somewhat nervous and hysterical, but much less so than I had a right to expect from the excitement and fatigue to which she had been subjected, and than more trivial circumstances had induced on many previous occasions. She was perfectly intelligent; there was no drowsiness; and nothing approaching to the "anæsthetic condition" presented itself at that time, or afterwards. Some whiskey was administered, and she seemed to be doing well.

Just at this period the young lady who had accompanied her to the dental office, and who had been greatly interested and excited by what had occurred there, fell upon the floor in a violent paroxysm of hysteria. As her health was delicate and such attacks unusual, I was extremely solicitous on her account, and, for some time gave my attention exclusively to her.

After she had been removed to another chamber, placed in bed, and morphia administered subcutaneously, I went to look after my first patient, and found, to my great regret, that the additional excitement had been too much for her, and that one of her regular "attacks" had devoloped itself in the interval. She was still sensible—so much so as to be able to talk about her condition, and to assist in undressing herself—but the hysterical symptoms were momentarily growing more violent, and an acute pain locating itself alternately in the uterus and heart, was complained of.

One-quarter of a grain of morphia in solution was then injected into her arm, at her solicitation—the pulse never having been depressed by the action of the chloroform. Two hours and a-half after the use of this remedy, as the pain had increased, and there was neither abatement of the nervous symptoms nor the least disposition to somnolency, the same quantity of the narcotic—one-quarter of a grain in solution—was again employed in a similar manner.

Other engagements called me soon afterwards from the house, but I learn that she gradually became more composed, and finally slept. Her friends inform me that there was no stertorous breathing, no unnatural slowness or irregularity of pulse, no alterations of the countenance, no dampness and coldness of the surface, and in fact, none of those peculiar symptoms by which *narcotism* is so infallibly indicated, and so readily diagnosed.

About half-past seven in the evening, more than eight hours after the use of the chloroform, her friends were startled by a gurgling sound with slight struggling, and hastening to her room, found her in a sinking condition.

Professor H. L. Byrd, who resides in the immediate neighborhood, was hastily summoned, and in vain attempted to revive her. Under his judicious treatment her pulse rallied for a few moments, but sank again into the profound quiescence of death. I reached the room just as the last expiration released her lovely spirit from its earthly tenement and sent it forth on its heavenward journey. Her extremities were still warm; her countenance was tranquil and natural; her pupils were not contracted; and every thing indicated that death had been sudden, and that it had commenced at the heart.

Three questions naturally suggest themselves, in this connection, which require satisfactory answers.

I. Why was chloroform administered at all, to a patient suffering from disease of the heart?

To this I would respond, *firstly,* that the medical attendant, after exhausting argument and entreaty, was constrained to yield to the wishes of the young lady, in order to prevent the administration of the anæsthetic by an inexperienced hand, and that it *might be properly done*:

*Secondly,* That the disease in question was not structural or organic, but exclusively nervous or functional—a species of angina pectoris—and that, according to the teachings of every man of recognized position and authority, in the profession, chloroform is *par excellence* the appropriate remedy for such affections: and, *thirdly,* that on many previous occasions, both with my sanction and without it, she had used this agent with positive impunity.

II. Was the patient's fate hastened by the remedies employed?

The answer to this question is equally plain and satisfactory. Chloroform produces its toxic effects by overwhelming the medulla oblongata, and thus paralyzing the organs and suspending the functions which are presided over by that great centre. This result, from the very nature of things, is never postponed, but is instantaneous. The patient dies while under the immediate influence of the drug—death takes place upon the table while anæsthesia exists. The heart ceases to beat at that period when the medulla is most under the control of this subtle but powerful agent. The danger is directly proportional to the profundity and persistence of the anæsthetic condition, and inversely so to the rapidity and completeness of the recovery—to the return to consciousness, feeling and mobility.

Nor does anæsthesia reproduce itself. Its subsidence is a finality. Each moment serves to eliminate from the blood more and more of the agent upon which this peculiar condition depends, and to increase the difficulties of its re-development.

In this instance, the patient passed safely through the period of danger—neither heart, lungs, nor brain were injuriously impressed by the agent. Her recovery was rapid and complete. No sign or symptom—not the slightest trace or indication—of the anæsthetic condition was developed at any subsequent period in the history of the case, and sufficient time—more than eight hours—had elapsed to preclude the contingency of its re-appearance.

Again, the observations of medical men throughout the world, have established, that however dangerous it may be in its *primary* influence upon the economy, its *secondary* effect is to induce repose, rest, tranquility throughout the nervous system. In the Italian campaign of 1859, this fact was demonstrated in thirty thousand instances, there being no exception to the general rule. Mr. McLeod, in his Surgical Notes on the Crimean War, reports twenty thousand cases in which the same truth was exemplified. My own experience, together with that of multitudes of surgeons who served on either side in the late struggle, sustains this conclusion to the fullest and farthest extent.

The previous history of Miss Jones' case convinces me that some tranquilizing but not depressing influence was exerted upon her by the secondary action of the chloroform, as she was much less nervous up to the attack of her friend than she had frequently been made by the most trivial circumstance when no such restraining power was in operation.

So far as the hypodermic use of the morphia is concerned the whole matter is readily disposed of.

She had repeatedly taken as much as a *whole grain* by this method, with impunity; the dose was too small a one to produce injurious effects *under any circumstances,* and her condition immediately preceding and succeeding her death, furnishes the most incontestable proof that she was *not narcotised.*

III. Did she really die of disease of the heart?

It has been demonstrated that neither the chloroform nor the morphia was instrumental in bringing about the sad termination. It has, also, been shown that she suffered at times from a most painful and dangerous affection of her heart—from a disease of that organ which had repeatedly placed her life in jeopardy. These circumstances, taken in conjunction with the suddenness of her demise, with the intense pain previously experienced in the præcordial region, and with the condition of the body after death, her warm extremities, natural countenance, dry skin, normal pupils, &c., point with unerring certainty to the heart as the point from which the fatal shaft was hurled.

Apart from these *a priori* reasonings, this position is rendered impregnable by the testimony of the only competent witness to the closing scene of this unfortunate lady's life. Professor Byrd, whose ability to form a correct judgment cannot be questioned, and to whom alone was furnished an opportunity to arrive at an intelligent conclusion in this connection, gives it as his unqualified belief that she "died of disease of the heart." His written opinion is appended to this paper. My own convictions fully accord with his; and I feel assured that such will be the deliberate judgment of any intelligent and honest professional man who carefully considers the facts embodied in this article.

I have, therefore, no hesitation in announcing that Miss Jones died of *cardiac neuralgia* incident to an attack of hysteria—a disease from which she had suffered intensely during a great portion of her brief existence.

The following statements will, without doubt, place the whole matter in its proper light before both professions.

---

STATEMENT OF PROFESSOR GORGAS.

BALTIMORE, *April* 15, 1867.

PROF. EDWARD WARREN—*Dear Sir:*

I have carefully read the statements contained in the above article, and take pleasure in bearing the most emphatic testimony to their correctness, so far as they relate to what transpired in my presence.

I had declined to administer chloroform in the case of Miss Jones, for the reason, that she informed me that she was subject to a serious *affection of the heart.* Of the nature of that disease, I had not enjoyed an opportunity to inform myself; and my objections to taking the responsibility of using the anæsthetic, were based upon the general rule which has been established on this subject.

On the 28th of March last Miss Jones called at my office with you, and again insisted upon taking chloroform.

In response to our united efforts to dissuade her from so doing, she replied positively, that *she would take the chloroform if it killed her*, and that, if *you refused to comply with her request she would have it administered by some one else.*

"Richardson's Spray Apparatus" was then tried, but so soon as the ether came in contact with her mouth, she refused to submit to the application, but again declared she would have chloroform.

Entreaty was once more attempted but in vain. The dangers incident to the inhalation of chloroform in an erect position were presented to her, without effect; you then administered the chloroform, and I extracted the tooth. She rapidly recovered from the effect of this agent, and in about half an hour, left my office apparently perfectly well. In response to your persuasion to remain longer, or at least to ride home in a hack, she replied that she was "going to Preston street to see a relative," and actually ran, laughing and talking, out of the door in advance of her friends, to all appearances, in as good health as she ever was. I regarded her recovery from the anæsthetic state as complete.

An experience of ten years in the use of anæsthetic agents enables me to say, that a more careful and skilful administration of chloroform could not have been made. A very small quantity of the agent was used, and every possible precaution that science and experience could suggest was taken to prevent a fatal result.

I have no idea that the chloroform had anything to do with her death, but am convinced that she died from "disease of the heart."

Yours, very truly, &c.

F. J. S. GORGAS, M.D.,

*Prof. of Dental Surgery in Baltimore College.*

---

STATEMENT OF PROF. BYRD.

*No.* 21 *North Calvert Street.*

BALTIMORE, *April* 4, 1867.

PROF. EDWARD WARREN—*My Dear Doctor:*

In response to your request for an expression of my professional opinion in regard to the cause of the death of Miss E. J. Jones, I have to say that it is my belief that she died *from disease of the heart.*

Very respectfully, your obe't servant,

H. L. BYRD, M.D

---

